# Correlation between TyG index, TyG-BMI index and AIP index and early-onset type 2 diabetic nephropathy

**DOI:** 10.3389/fendo.2026.1778646

**Published:** 2026-04-10

**Authors:** Xiangyan Wei, Baolan Liang, Airong Chen

**Affiliations:** 1 The Second Clinical Medical School, Lanzhou University, Lanzhou, Gansu, China; 2 Department of Endocrinology and Metabolism, The Second Hospital of Lanzhou University, Lanzhou, Gansu, China

**Keywords:** atherogenic index of plasma, diabetic nephropathy, early onset type 2 diabetes, insulin resistance, triglyceride-glucose index, triglyceride-glucose-body mass index

## Abstract

**Objective:**

To explore and compare the correlation between triglyceride-glucose (TyG) index, triglyceride-glucose body mass index (TyG-BMI) and plasma atherogenic (AIP) index and early onset type 2 diabetic nephropathy.

**Methods:**

179 patients with early-onset type 2 diabetes admitted to the Department of Endocrinology, The Second Hospital of Lanzhou University from January 2024 to June 2025 were selected retrospectively. According to the ratio of urinary microalbumin to creatinine (UACR), they were divided into early-onset T2DM group (UACR<30mg/g) and DKD group (UACR≥30mg/g). Spearman correlation was performed to analyze the correlation between insulin resistance parameters and UACR, Logistic regression was used to analyze the risk factors of DKD, and the curve of ROC was drawn to evaluate the predictive efficacy of TyG index, TyG-BMI index and AIP index.

**Results:**

There were 129 cases (72.07%) in T2DM group and 50 cases (27.93%) in DKD group. The levels of age, course of disease, TG, UTP, UACR, HOMA-IR, TyG, TyG-BMI, AIP and TC/HDL-C in early-onset T2DM with DKD were significantly compared to that of early-onset T2DM (P < 0.05). TyG, TyG-BMI, AIP, and TC/HDL-C were positively correlated with UACR (P<0.05). TyG, TyG-BMI and AIP were independent risk factors for early-onset T2DM complicated with DKD (P<0.05). Additionally, TyG, TyG-BMI and AIP are all effective indicators for predicting DKD in patients with early-onset T2DM. The order according to the area under the curve (AUC) is: TyG index (AUC 0.690, 95%CI 0.606-0.773), TyG-BMI index (AUC 0.600, 95%CI 0.506-0.695), AIP index (AUC 0.678, 95%CI 0.595-0.761). Among the three indices, the TyG index showed a relatively stronger association with DKD (AUC 0.690), though its discriminative ability remains modest. These indices may serve as adjunctive screening tools rather than standalone diagnostic tests for early-onset DKD, and its optimal critical value for predicting DKD is 10.0013.

**Conclusion:**

The increase of TyG, TyG-BMI and AIP index are associated factors for patients with early-onset type 2 diabetic nephropathy. TyG index has the strongest diagnostic value, the best sensitivity and the strongest correlation with UACR, and can be used as the most valuable IR substitute index for DKD risk prediction in early-onset T2DM patients.

## 1 Introduction

In recent years, the global incidence of diabetes has continued to rise, with over 90% of cases being type 2 diabetes, and a trend toward younger age groups (1). Early-onset type 2 diabetes (EOD) refers to T2DM diagnosed before age 40. According to the International Diabetes Federation report, the prevalence of type 2 diabetes among individuals aged 20–39 has increased by 30% globally, with the Middle East and North Africa region experiencing the largest relative increase (4% to 8%) (2). Compared to late-onset T2DM, EOD patients face more severe insulin resistance, higher risks of microvascular complications, and earlier mortality (3). Diabetic kidney disease (DKD) is one of the most common complications in T2DM patients, with mortality risks second only to cardiovascular disease and cancer. It has been reported that individuals with early-onset T2DM not only develop kidney disease earlier and experience faster progression but also represent an independent risk factor for end-stage kidney disease (ESKD) (4). Therefore, early identification of DKD is crucial for preventing and improving the prognosis of early-onset T2DM. Urinary Albumin/Creatinine Ratio (UACR) and estimated Glomerular Filtration Rate (eGFR), as two commonly used clinical indicators, still exhibit insufficient sensitivity and specificity in the early stages of kidney injury. Consequently, clinical practice necessitates the discovery and validation of earlier, more precise risk prediction markers.

Studies have shown that insulin resistance is closely associated with the onset and progression of early-onset T2DM and DKD. Research by Myrsini et al. (5) indicates that patients with early-onset T2DM commonly exhibit overweight or obese conditions, leading to extensive ectopic fat deposition in organs such as the liver. This triggers systemic inflammation, exacerbates insulin resistance, and thereby accelerates disease onset and progression. Insulin resistance promotes renal cell hypertrophy and tissue remodeling by disrupting insulin signaling pathways and activating growth pathways. Simultaneously, it compromises the glomerular filtration barrier, leading to proteinuria and impaired renal function, thereby accelerating the progression of kidney damage (6). Although the hyperinsulinemic-euglycemic clamp technique is the gold standard for assessing insulin resistance, its limited reproducibility and invasive, complex nature make it difficult to implement clinically. The Homeostasis Model Assessment of Insulin Resistance (HOMA-IR) is a commonly used indicator for evaluating insulin resistance severity. However, HOMA-IR has some limitations due to the variations in insulin detection methods and the influence of exogenous insulin. Simpler, low-cost surrogate markers for insulin resistance are more clinically applicable. For instance, the Triglyceride-Glucose Index (TyG) combines fasting triglycerides and glucose levels to assess insulin resistance and metabolic syndrome, showing significant positive correlation with UACR (7). The Triglyceride-Glucose-Body Mass Index (TyG-BMI) incorporates body mass index, enhancing predictive power for diabetes risk and cardiovascular events (8); The Atherogenic Index of Plasma (AIP), based on the ratio of triglycerides to high-density lipoprotein cholesterol, is closely associated with atherosclerotic risk and insulin resistance (9). Current research lacks studies on the correlation between insulin resistance parameters and early-onset type 2 with diabetic kidney disease (DKD). Therefore, this retrospective analysis of clinical data from 179 patients with early-onset T2DM aims to evaluate the predictive value of different insulin parameters for early-onset T2DM complicated by DKD, providing targeted evidence for early screening and intervention in this population.

## 2 Methodology

### 2.1 Participants

A retrospective cohort study of 179 patients with early-onset type 2 diabetes mellitus (T2DM) hospitalized in the Department of Endocrinology at Lanzhou University Second Hospital from January 2024 to June 2025 was selected. The cohort comprised 147 males and 32 females, with a median age of 35 (31, 38) years. Patients were grouped based on UACR ratio results: those with <30 mg/g were assigned to the early-onset T2DM group (n=129), while those with ≥30 mg/g were assigned to the early-onset T2DM with DKD group (n=50). Inclusion criteria: (1) All subjects aged ≥18 years. (2) Patients with early-onset type 2 diabetes defined as <40 years old according to the American Diabetes Association (ADA) and World Health Organization (WHO) criteria (10). (3) Patients diagnosed with DKD based on the KDIGO 2020 Clinical Practice Guideline for the Management of Chronic Kidney Disease in Diabetes (11). (4) Complete medical records. Exclusion criteria: (1) Diabetes types other than T2DM, such as type 1 diabetes or special types of diabetes; (2) Patients with acute diabetic complications; (3) Other renal diseases besides DKD, such as acute/chronic glomerulonephritis or nephrotic syndrome; (4) Patients undergoing hemodialysis, peritoneal dialysis, or kidney transplantation; (5) Patients with severe psychiatric or physical illnesses, such as malignant tumors, severe hepatic insufficiency, or severe psychiatric disorders; (6) Patients suspected of having familial hypertriglyceridemia. This study was approved by the Ethics Committee of the Second Hospital of Lanzhou University (Approval No.: 2025A-1340). All patients provided informed consent.

### 2.2 Methods

Clinical Data Collection Collect general information including gender, age, disease duration, height, weight, body mass index (BMI), and blood pressure for all patients.

Laboratory Indicator Testing Venous blood samples collected from all patients the morning after admission, following an 8-hour fasting period, were sent to the Laboratory Department of Lanzhou University Second Hospital. Biochemical indicators were measured using a Hitachi 7600-DDP fully automated biochemical analyzer, including fasting plasma glucose (FPG), total cholesterol (TC), triglycerides (TG), high-density lipoprotein cholesterol (HDL-C), low-density lipoprotein cholesterol (LDL-C), serum creatinine (SCr), and serum uric acid (UA). Midstream urine samples were collected the following morning to determine the urinary albumin-to-creatinine ratio (UACR). Urinary albumin was measured by immunoturbidimetry, and urinary creatinine by enzymatic method (Hitachi 7600-DDP automatic analyzer.

Insulin resistance parameters were calculated as follows: TyG = Ln[TG(mg/dL) × FPG(mg/dL)/2]; TyG-BMI = Ln[TG(mg/dL) × FPG(mg/dL)/2] × BMI; AIP = Log[TG(mmol/L)/HDL-C(mmol/L)]; HOMA-IR = FPG(mmol/L) × FINS(μU/mL)/22.5.

To ensure the accuracy of the early-onset T2DM diagnosis, we rigorously reviewed medical records. All patients diagnosed before age 20 underwent additional screening to rule out monogenic diabetes (e.g., MODY) and type 1 diabetes. This screening included the absence of islet autoantibodies (GAD, IA-2), measurable C-peptide levels (>0.2 nmol/L) at diagnosis, and no immediate requirement for insulin therapy upon diagnosis. Patients with suspected familial hypertriglyceridemia or atypical forms of diabetes were excluded based on the exclusion criteria. Information regarding the use of medications that could potentially affect UACR (such as ACE inhibitors/ARBs) or lipid profiles (such as statins and fibrates) at the time of admission was not available for all patients due to the retrospective nature of the data collection. Information regarding the use of medications that could potentially affect UACR (such as ACE inhibitors/ARBs) or lipid profiles (such as statins and fibrates) at the time of admission was not available for all patients due to the retrospective nature of the data collection.

### 2.3 Statistical analysis

Data processing and statistical analysis were performed using the R programming language. Data normality was assessed using the Shapiro-Wilk test. Normally distributed quantitative data were expressed as mean ± standard deviation and compared between groups using the independent samples t-test. Non-normally distributed quantitative data were presented as median and interquartile range (IQR), and comparisons between groups were performed using the nonparametric rank-sum test. Categorical data were reported as (n, %) and compared between groups using the chi-square test. Spearman correlation analysis was used to describe relationships between variables, with correlation coefficients denoted by r. Multivariate logistic regression analyzed factors influencing early-onset T2DM with DKD. Receiver operating characteristic (ROC) curves were plotted to calculate area under the curve (AUC), sensitivity, specificity, and cutoff values. Differences were considered statistically significant at P < 0.05. To avoid model overfitting due to the limited number of DKD events (n=50), we adhered to the principle of having at least 10 events per variable (EPV). Given the potential for multicollinearity among the insulin resistance surrogates (TyG, TyG-BMI, AIP), we did not include them simultaneously in a single multivariate model. Instead, we constructed three separate multivariate logistic regression models, each adjusting for the same core confounders (age, duration, and UTP) to assess the independent effect of each index (TyG, TyG-BMI, and AIP) individually. This approach ensures an adequate EPV and minimizes the risk of overfitting. Given the exploratory nature of this study, comparisons of baseline characteristics between groups in **Table 1** were performed without adjustment for multiple testing. The P-values presented in **Table 1** are considered descriptive and hypothesis-generating rather than definitive confirmatory evidence. The primary conclusions of this study regarding the association of TyG, TyG-BMI, and AIP with DKD are based on the multivariable logistic regression analyses (which were conducted separately for each index to mitigate collinearity and overfitting) and the pre-specified correlation analyses.

**Table 1:** Comparison of clinical data between the group with pure early-onset type 2 diabetes and the group with combined DKD.

Parameter	Simple early-onset T2DM group (n = 129)	Merged DKD group DKD (n = 50)	Statistics	P value
Gender, n(%)			χ²=0.00	0.979
Male	106 (82.17)	41 (82.00)		
Female	23 (17.83)	9 (18.00)		
Age, M (Q_1_, Q_3_)	35.00 (31.00, 37.00)	37.00 (34.00, 38.75)	Z=-2.51	0.012
Duration(月), M (Q_1_, Q_3_)	12.00 (2.00, 48.00)	54.00 (11.25, 96.00)	Z=-3.69	<0.001
Height(cm), M (Q_1_, Q_3_)	172.00 (168.00, 178.00)	171.50 (170.00, 176.00)	Z=-0.22	0.829
Weight(kg), M (Q_1_, Q_3_)	78.00 (68.00, 85.00)	78.25 (66.50, 90.00)	Z=-0.89	0.371
BMI, M (Q_1_, Q_3_)	25.71 (23.89, 28.32)	25.97 (23.19, 29.17)	Z=-0.48	0.634
SBP(mmHg), M (Q_1_, Q_3_)	124.00 (113.00, 135.00)	124.50 (115.25, 131.75)	Z=-0.08	0.938
DBP(mmHg), M (Q_1_, Q_3_)	82.00 (75.00, 90.00)	82.00 (76.25, 92.75)	Z=-0.41	0.682
Hypertension, n(%)			χ²=3.46	0.063
No	84 (65.12)	25 (50.00)		
Yes	45 (34.88)	25 (50.00)		
FBG(mmol/L), M (Q_1_, Q_3_)	11.41 (7.93, 15.36)	13.43 (8.64, 15.65)	Z=-1.38	0.166
FINS(mU/L), M (Q_1_, Q_3_)	11.90 (6.74, 19.70)	15.23 (7.95, 21.40)	Z=-1.29	0.197
FCP(ng/ml), M (Q_1_, Q_3_)	2.13 (1.58, 3.10)	2.79 (1.64, 3.85)	Z=-1.59	0.113
2hPG(mmol/L), M (Q_1_, Q_3_)	10.93 (8.76, 13.74)	11.95 (9.73, 13.26)	Z=-0.20	0.839
2hINS(mU/L), M (Q_1_, Q_3_)	31.97 (16.24, 56.12)	29.01 (19.52, 62.58)	Z=-0.13	0.898
2hCP(ng/ml), M (Q_1_, Q_3_)	3.95 (2.64, 5.56)	3.51 (2.13, 6.36)	Z=-0.16	0.875
HbA1c(%), M (Q_1_, Q_3_)	9.80 (7.90, 11.50)	10.60 (7.72, 11.50)	Z=-0.41	0.678
TC(mmol/L), M (Q_1_, Q_3_)	4.77 (4.15, 5.36)	5.03 (4.40, 5.89)	Z=-2.00	0.046
TG(mmol/L), M (Q_1_, Q_3_)	2.01 (1.23, 3.37)	3.28 (2.11, 4.95)	Z=-3.77	<0.001
HDL-C(mmol/L), M (Q_1_, Q_3_)	1.03 (0.88, 1.22)	0.99 (0.89, 1.18)	Z=-0.82	0.410
LDL-C(mmol/L), Mean ± SD	3.07 ± 0.71	3.22 ± 0.82	t=-1.24	0.218
VitD(ng/ml), M (Q_1_, Q_3_)	6.81 (4.80, 9.42)	6.78 (4.54, 9.72)	Z=-0.11	0.915
iPT(pg/ml), M (Q_1_, Q_3_)	39.60 (25.50, 50.70)	34.40 (22.25, 48.90)	Z=-0.87	0.384
BUN(mmol/L), M (Q_1_, Q_3_)	5.32 (4.29, 6.26)	5.46 (4.85, 6.84)	Z=-1.81	0.070
SCr(μmol/L), M (Q_1_, Q_3_)	59.50 (50.70, 68.40)	59.15 (52.05, 74.83)	Z=-0.97	0.330
UA(μmol/L), M (Q_1_, Q_3_)	356.00 (283.00, 439.00)	380.00 (309.25, 416.75)	Z=-1.09	0.276
eGFR(ml/min/1 73m2), M (Q_1_, Q_3_)	125.25 (117.00, 134.30)	122.72 (112.22, 129.31)	Z=-1.70	0.090
UTP(g/24h), M (Q_1_, Q_3_)	0.18 (0.12, 0.22)	0.34 (0.21, 0.97)	Z=-6.85	<0.001
UACR(mg/g), M (Q_1_, Q_3_)	8.84 (6.19, 11.49)	95.03 (52.16, 341.00)	Z=-10.38	<0.001
HOMA-IR, M (Q_1_, Q_3_)	5.34 (3.50, 10.06)	8.29 (4.14, 12.48)	Z=-1.97	0.049
TyG, Mean ± SD	9.85 ± 0.97	10.42 ± 0.86	t=-3.61	<0.001
TyG-BMI, Mean ± SD	256.80 ± 49.41	279.12 ± 59.69	t=-2.55	0.012
AIP, M (Q_1_, Q_3_)	0.29 (0.10, 0.56)	0.50 (0.30, 0.75)	Z=-3.69	<0.001
TC/HDL-C, M (Q_1_, Q_3_)	4.61 (3.77, 5.30)	5.11 (4.53, 5.98)	Z=-2.57	0.010
LDL-C/HDL-C, M (Q_1_, Q_3_)	3.05(2.35, 3.57)	3.33(2.89, 3.74)	Z=-1.91	0.057

P values are descriptive and were not adjusted for multiple comparisons.

## 3 Results

### 3.1 Participant characteristics

This study included 179 patients with early-onset T2DM aged 18–40 years. Males constituted 82.1% (147/179), while females accounted for 17.9% (32/179). The median age was 35 (31, 38) years, the median diabetes duration was 24 (3, 72) months, and the median BMI was 25.73 (23.61, 28.39) kg/m². The prevalence of DKD among patients with early-onset T2DM was 27.93% (50/179).

### 3.2 Comparison of clinical data between the two groups

Among the 179 patients with early-onset T2DM in this study (**Table 1**), 129 (72.07%) belonged to the early-onset T2DM group, while 50 (27.93%) had DKD. Compared with the T2DM group, both groups exhibited significantly higher levels of age, disease duration, TG, UTP, UACR, HOMA-IR, TyG, TyG-BMI, AIP, and TC/HDL-C, with all differences being statistically significant (all P < 0.05). No statistically significant differences were observed between the two groups in height, weight, BMI, SBP, DBP, FBG, FINS, FCP, 2hPG, 2hINS, 2hCP, HbA1c, BUN, SCr, UA, TC, HDL-C, LDL-C, VitD, iPT, eGFR, or LDL-C/HDL-C (P > 0.05).

### 3.3 Correlation analysis between DKD renal impairment and insulin resistance parameters

As shown in **Table 2**, UACR in patients with early-onset T2DM was positively correlated with TyG, TyG-BMI, AIP, TC/HDL-C (P<0.05).

**Table 2:** Correlation analysis of UACR with insulin resistance parameters.

Index	R value	P value
TyG	0.383	<0.001
TyG-BMI	0.162	0.030
AIP	0.318	<0.001
HOMA-IR	0.046	0.538
TC/HDL-C	0.163	0.030
LDL-C/HDL-C	0.113	0.132

### 3.4 Analysis of risk factors for DKD development in patients with early-onset T2DM

Early-onset T2DM with or without DKD was analyzed as the dependent variable (1=with DKD, 0=without DKD), with TyG, TyG-BMI, and AIP as independent variables. Results indicated that TyG, TyG-BMI, and AIP were all independent risk factors for DKD in patients with early-onset T2DM (P<0.05). See **Table 3**.

**Table 3:** Univariate and multivariate Logistic regression analysis of early-onset type 2 diabetes mellitus combined with diabetic kidney disease.

Variables	Single-factor					Multi-factor				
	β	S.E.	Wald χ²	P	OR (95%CI)	β	S.E.	Wald χ²	P	OR (95%CI)
TyG	0.62	0.18	11.86	<0.001	1.86 (1.30-2.68)	0.58	0.23	6.36	0.013	1.78 (1.13-2.82)
TyG-BMI	0.01	0.00	6.10	0.013	1.01 (1.01-1.01)	0.01	0.00	4.41	0.036	1.01 (1.01-1.02)
AIP	1.44	0.44	10.71	<0.001	4.20 (1.79-9.87)	1.34	0.53	6.39	0.011	3.82 (1.35-10.76)
Age	0.07	0.04	4.24	0.039	1.08 (1.01-1.15)					
Duration	0.02	0.00	16.32	<0.001	1.02 (1.01-1.02)					
UTP(g/24h)	8.62	1.84	21.95	<0.001	5531.44 (151.26-202279.67)					

### 3.5 Predictive performance of TyG, TyG-BMI, and AIP for early-onset T2DM with DKD

**Figure 1:**
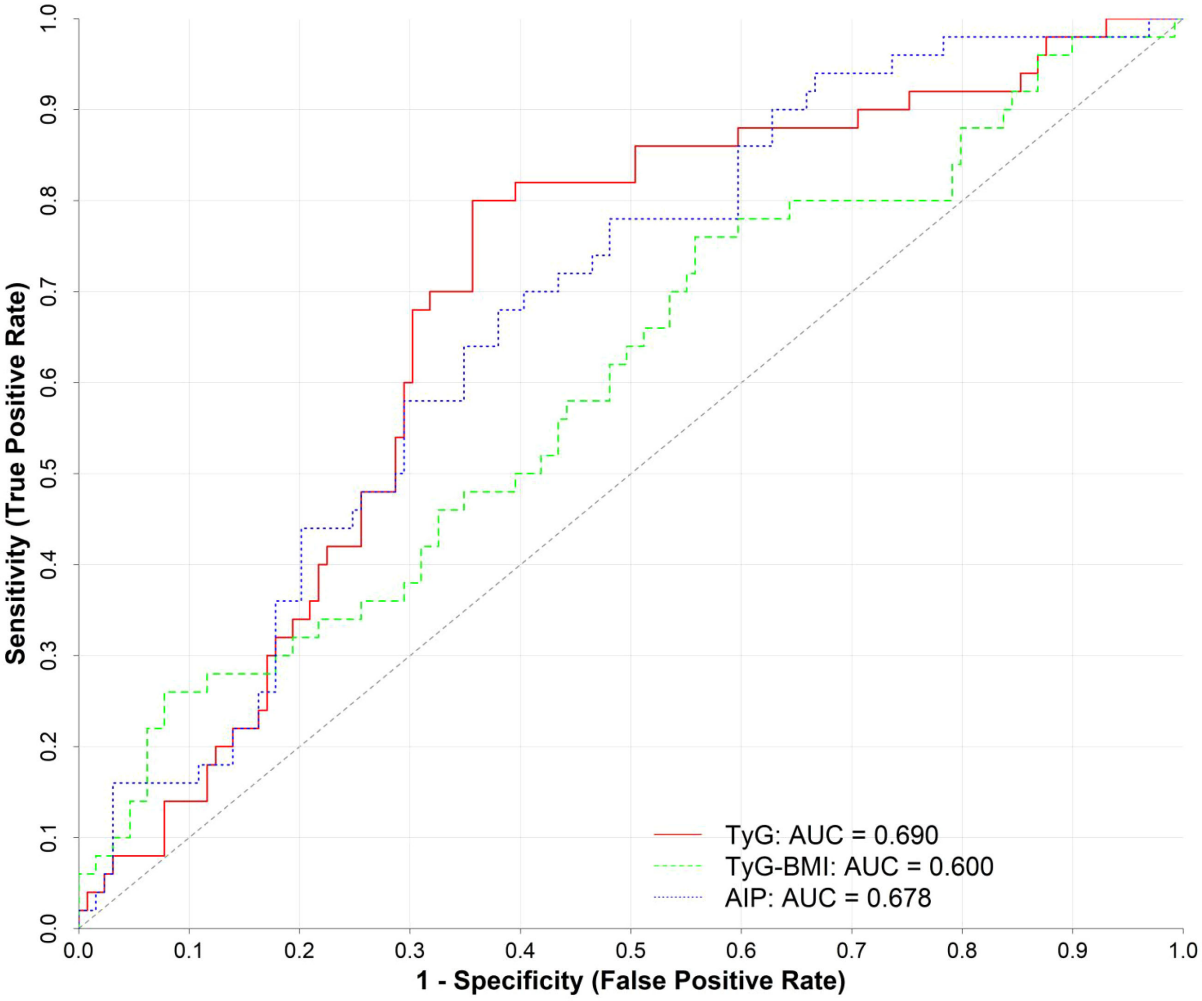
ROC curves for TyG, TyG-BMI, and AIP in predicting early-onset type 2 diabetes mellitus combined with DKD.

ROC curve analysis was performed using TyG, TyG-BMI, and AIP levels as test variables and the presence of DKD as the state variable. Results indicate that TyG, TyG-BMI, and AIP all possess predictive value for DKD development, with AUC values of 0.690, 0.600, and 0.678, respectively. Optimal cutoff values were determined as 10.0013, 242.9289, and 0.3787 (**Table 4**; **Figure 1**).

**Table 4:** Efficacy analysis of TyG, TyG-BMI, and AIP in the prevention of early-onset type 2 diabetes mellitus combined with DKD.

Variables	AUC	95%CI	Cutoff value	Sensitivity	Specificity	YI
TyG	0.690	0.606-0.773	10.0013	0.800	0.643	0.443
TyG-BMI	0.600	0.506-0.695	242.9289	0.760	0.442	0.202
AIP	0.678	0.595-0.761	0.3787	0.680	0.620	0.300

## 4 Discussion

The global prevalence of diabetes is on the rise. As a major microvascular complication of T2DM, DKD is not only the leading cause of end-stage renal disease but is also closely associated with significantly elevated morbidity and mortality risks (12). Studies indicate that between 2012 and 2023, the incidence of T2DM among young populations increased by 46% to 71%, with a concurrent significant rise in microvascular complications, particularly diabetic neuropathy and nephropathy (13). Our findings indicate that among 179 early-onset T2DM cases, males predominated. Of these, 50 (27.93%) had DKD, while 129 (72.07%) had T2DM. This suggests a higher DKD incidence in early-onset T2DM patients, highlighting the critical importance of studying risk factors for early-onset T2DM with DKD for timely prevention and treatment. Additionally, it was found that hyperglycemia, hyperlipidemia, hyperinsulinemia, weight gain, and vitamin D deficiency were prevalent in early-onset T2DM, consistent with previous reports (4, 14, 15). We acknowledge the notable male predominance (82.1%) in our study cohort. This imbalance may reflect a combination of factors: first, the demographic characteristics of the early-onset T2DM population in our region; second, potential selection bias inherent to a single-center study from a department where male patients might have higher hospitalization rates. Consequently, our findings may have limited generalizability to female patients with early-onset T2DM, and further studies with a more balanced sex distribution are warranted. While no significant differences were observed between the two groups in BMI, glycated hemoglobin, insulin, and other laboratory indicators, lipid levels were higher in early-onset T2DM patients with DKD. Thongnak L et al. (16) suggested that renal lipotoxicity might be a key factor in DKD progression, where accumulated renal lipids and their metabolites induced oxidative stress, inflammation, and fibrosis, further impairing renal function. Moreover, both age and disease duration were higher in the early-onset T2DM with DKD group compared to the T2DM group, representing risk factors for DKD development.

Recent studies indicate that individuals with early-onset T2DM with DKD exhibit a certain degree of insulin resistance (IR). Besides, those with lipid metabolism abnormalities (such as elevated triglycerides or low HDL-C) may demonstrate significant insulin resistance (17). Furthermore, lipid-related insulin resistance parameters outperform conventional lipid biochemical indicators in assessing metabolic abnormality risk. Here, the TyG, TyG-BMI, AIP index, and TC/HDL-C ratio were significantly higher in the early-onset T2DM with DKD group compared to the T2DM-only group, and all parameters were positively correlated with UACR. To assess whether TyG, TyG-BMI, and AIP are risk factors for DKD, separate multivariate logistic regression models were employed to avoid multicollinearity. Univariate analysis revealed that age, disease duration, UTP, TyG index, TyG-BMI index, and AIP index were all closely associated with the risk of DKD development. After adjusting for age, disease duration, and UTP—which were significant in univariate analysis—multivariate logistic regression analysis further identified TyG, TyG-BMI, and AIP index as independent risk factors for early-onset T2DM with DKD. ROC curve analysis demonstrated that TyG, TyG-BMI, and AIP are effective indicators for predicting early-onset T2DM with DKD. Among these, the TyG index exhibited superior diagnostic performance compared to other indicators (AUC = 0.690, P < 0.05).

TyG index, a simple, cost-effective, and easily applied alternative marker to IR, was calculated based on FPG and TG levels and could be used to screen for microvascular complications in T2DM patients (18). Chiu H et al. (19) found that a higher TyG index in T2DM patients was associated with microalbuminuria, suggesting the TyG index may serve as a potential risk marker for microvascular complications in type 2 diabetes patients. Liu et al. (20) investigated the correlation between the TyG index and microvascular complications in patients with early-onset T2DM. They found that the TyG index in early-onset T2DM patients was positively correlated with the severity of diabetic nephropathy (DN), and the TyG index was an independent risk factor for diabetic nephropathy (OR = 1.623, 95% CI = 1.175-2.242). Elevated TyG index reflects more severe insulin resistance, which can accelerate DKD progression by inducing metabolic disorders, enhancing oxidative stress, and promoting inflammatory responses, thereby causing renal damage. This study found that the TyG index was higher in the early-onset T2DM with DKD group than in the T2DM group (10.42 ± 0.86 vs. 9.85 ± 0.97, P < 0.001) and was positively correlated with UACR. After adjusting for confounding factors, the TyG index remained associated with an increased risk of DKD onset (OR = 1.78, 95% CI = 1.13-2.82, P<0.001). ROC curve analysis demonstrated that the TyG index possesses high predictive value for DKD (AUC = 0.690, sensitivity 80.0%, specificity 64.3%), with a cutoff value of 10.0013.

The TyG-BMI index, first proposed in 2016, outperforms traditional lipid ratios and is significantly associated with insulin resistance (21). A cross-sectional analysis using the US National Health and Nutrition Examination Survey (NHANES) database demonstrated that TyG-BMI is significantly correlated with DKD risk, with a marked increase in risk observed in the highest quartile (22). Jiang et al. (23) investigated the relationship between early-onset T2DM and the TyG-BMI index. In this work, higher TyG-BMI indices were associated with increased early-onset T2DM risk (P<0.001). ROC curve analysis demonstrated that TyG-BMI possesses good predictive value for diagnosing early-onset T2DM, with an AUC of 0.6781, outperforming the TyG index. In our study, the TyG-BMI index demonstrated lower ability than the TyG index to identify DKD in patients with early-onset T2DM (AUC 0.600, 95% CI 0.506–0.695), consistent with findings by Ke P et al. (24). The reason for this discrepancy may be that BMI, as a crude indicator of obesity, fails to distinguish the distribution of body fat. Furthermore, the presence of comorbidities such as hypertension and coronary heart disease may further accelerate the progression of kidney disease in obese individuals.

The AIP index is calculated by taking the logarithm of the TG to HDL-C ratio. This index can be used to identify atherogenic dyslipidemia and insulin resistance (25). The pro-atherogenic lipid environment represented by AIP deposits in the subendothelium of renal arteries, forming atherosclerotic plaques. These plaques cause luminal narrowing or even occlusion, reducing renal blood flow perfusion and ultimately leading to renal injury. Qi L et al. (26) investigated the predictive value of AIP for microalbuminuria in newly diagnosed T2DM patients. Results indicated that AIP is an independent risk factor for microalbuminuria, with an optimal cutoff value of 0.415 and an AUC of 0.722. In a study by Yan H et al. (27), AIP was significantly associated with the risk of DKD development and demonstrated predictive value for DKD incidence, with an optimal cutoff of 0.126 and an AUC of 0.592. This study found that the AIP index was higher in the early-onset T2DM with DKD group compared to the T2DM group, and it was positively correlated with UACR. AIP served as an independent risk factor for DKD renal injury and demonstrated high predictive value for DKD (AUC = 0.678, sensitivity 68.0%, specificity 62.0%), with a cutoff value of 0.3787. It is important to note that the AUC values for all three indices (ranging from 0.600 to 0.690) indicate only modest discriminative ability. This suggests that while these insulin resistance surrogates are significantly associated with DKD, they should not be used in isolation for clinical diagnosis. Rather, they may serve as complementary markers to existing clinical assessments, helping to identify high-risk patients who may benefit from closer monitoring or early intervention.

In summary, TyG, TyG-BMI, and AIP are all independent risk factors for early-onset T2DM patients with DKD and can indicate the occurrence and progression of DKD at an early stage. In clinical practice, monitoring these indicators and implementing early interventions may help reduce the risk of DKD in patients with early-onset T2DM. However, this study is a single-center retrospective analysis with limitations, including the absence of central obesity measurements, relatively small sample size affected by population heterogeneity, and lack of long-term DKD progression assessment in early-onset T2DM patients. Therefore, these findings require further validation through additional research.

Several limitations of this study should be acknowledged. First, due to its retrospective cross-sectional design, we can only establish an association between TyG, TyG-BMI, AIP and DKD, but cannot infer causality. It remains possible that the observed metabolic derangements are consequence, rather than a cause, of renal dysfunction. Therefore, our results should be interpreted as identifying correlative markers, not validated predictors, and prospective cohort studies are needed to confirm these findings. Second, as a single-center study conducted in a specific region of China, the generalizability of our findings to other populations may be limited. Multi-center studies with diverse ethnic and geographic populations are warranted. Finally, the multiple comparisons performed in the baseline characteristics table were not adjusted for, raising the potential for Type I errors. However, these analyses were intended to be descriptive, and our core findings are based on hypothesis-driven multivariable regression models. Furthermore, due to the retrospective design, data on medications that could influence UACR (e.g., ACE inhibitors/ARBs) or lipid profiles (e.g., statins, fibrates) were not available for the entire cohort. This lack of adjustment for concomitant medications may have introduced confounding bias. The direction and magnitude of this bias are uncertain, but it could potentially overestimate or underestimate the true associations. On the other hand, there is the lack of data on concomitant medications, particularly ACE inhibitors/ARBs and statins/fibrates. These drugs are known to directly influence both the outcome (UACR) and the exposure variables (TyG, AIP). The inability to adjust for these confounders by indication may have introduced bias, potentially overestimating or underestimating the true associations observed in our study. Although the male predominance (82.1%) limits generalizability to female patients, the small number of female participants (n=32) precluded meaningful sex-stratified analyses. Future studies with larger and more balanced cohorts are needed to explore potential sex-specific effects. Future prospective studies should collect detailed medication histories to account for this confounding.

## Data Availability

The original contributions presented in the study are included in the article/supplementary material. Further inquiries can be directed to the corresponding author.
